# Bioinformatics in China: A Personal Perspective

**DOI:** 10.1371/journal.pcbi.1000020

**Published:** 2008-04-25

**Authors:** Liping Wei, Jun Yu

**Affiliations:** 1 Center for Bioinformatics, National Laboratory of Protein Engineering and Plant Genetic Engineering, College of Life Sciences, Peking University, Beijing, People's Republic of China; 2 CAS Key Laboratory in Genome Sciences and Information, Beijing Institute for Genomics, Chinese Academy of Sciences, Beijing, People's Republic of China; University of California San Diego, United States of America

In this personal perspective, we recall the history of bioinformatics and computational biology in China, review current research and education, and discuss future prospects and challenges. The field of bioinformatics in China has grown significantly in the past decade despite a delayed and patchy start at the end of the 1980s by a few scientists from other disciplines, most noticeably physics and mathematics, where China's traditional strength has been. In the late 1990s and early 2000s, rapid expansion of the field was fueled by the Internet boom and genomics boom worldwide and in China. Today bioinformatics research in China is characterized by a great variety of biological questions addressed and the close collaborative efforts between computational scientists and biologists, with a full spectrum of focuses ranging from database building and algorithm development to hypothesis generation and biological discoveries. Although challenges remain, the future of bioinformatics in China is promising thanks to advances in both computing infrastructure and experimental biology research, a steady increase of governmental funding, and most importantly a critical mass of bioinformatics scientists consisting of not only converts from other disciplines but also formally trained overseas returnees and a new generation of domestically trained bioinformatics Ph.D.s.

## Introduction

**Figure 1:**
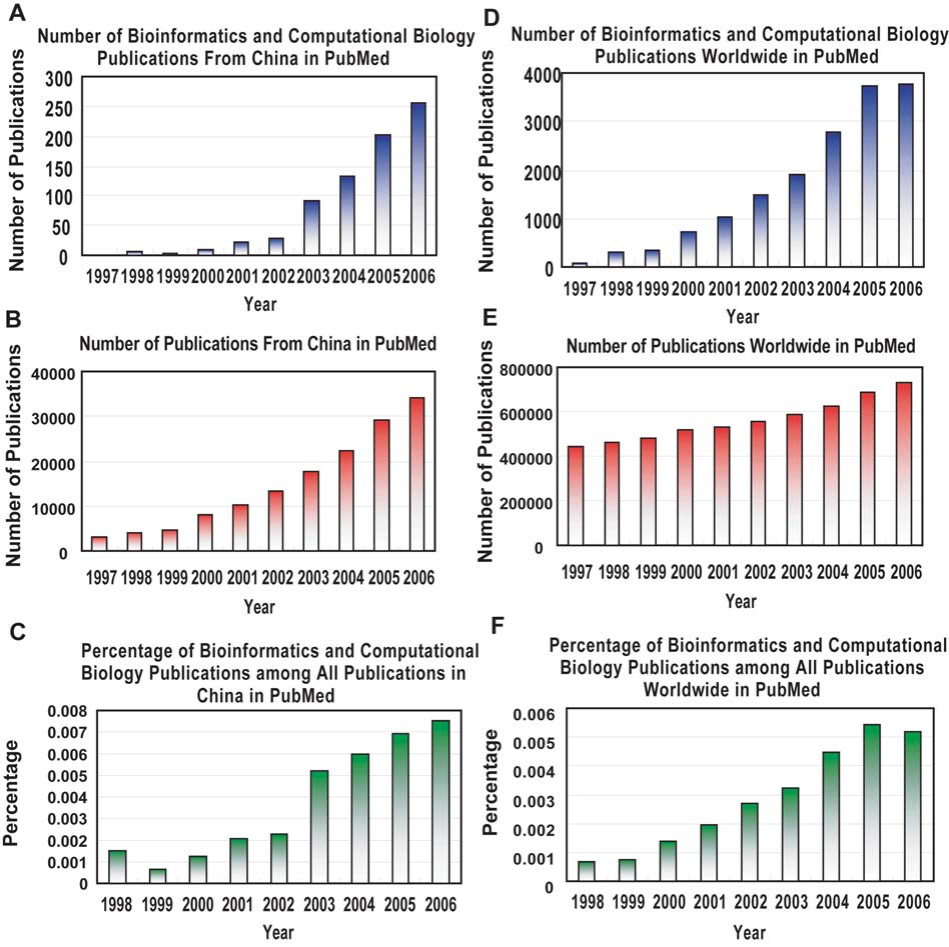
The number and percentage of bioinformatics publications from China and in all of PubMed in the past decade. (A) The number of bioinformatics and computational biology publications from China in PubMed, retrieved from NCBI by the Entrez query “China[affiliation] AND (bioinformatics OR computational biology)”. MESH has a heading “Computational Biology” of which “Bioinformatics” is an Entry Term. The numbers provide a rough indication of growth of the field. They tend to be underestimated because some bioinformatics publications cannot be retrieved by keywords “bioinformatics OR computational biology”. However adding more keywords would increase the false positive rate significantly. (B) The number of all publications from China in PubMed, retrieved from NCBI by the Entrez query “China[affiliation]”. (C) The percentage of bioinformatics publications in China among all publications in China, in PubMed. (D) The number of bioinformatics and computational biology publications in all of PubMed, retrieved from NCBI by the Entrez query “bioinformatics OR computational biology”. (E) The number of all publications in PubMed. (F) The percentage of bioinformatics publications among all publications, in PubMed.

The field of bioinformatics has enjoyed significant growth in China. A rough yet useful indicator of the field is the number of bioinformatics and computational biology publications from China indexed in PubMed at NCBI. As shown in Figure 1A, this number has been increasing significantly over the past decade. The number of all publications from China indexed in PubMed has also been increasing (Figure 1B), but if we plot the percentage of bioinformatics publications from China among all PubMed publications from China, we observe that the percentage itself has been increasing rapidly (Figure 1C), indicating that the contribution of bioinformatics research is becoming more significant within the life sciences in China. Comparing it with the situation worldwide, we observe that the number of bioinformatics publications from China is growing faster than the number of bioinformatics publications worldwide (Figure 1A versus 1D). Additionally, the number of PubMed publications from China has also been growing faster than the total number of PubMed publications (Figure 1B versus 1E). Furthermore, we observe that, very interestingly, for each year starting from 2003, the percentage of bioinformatics publications among all PubMed publications is greater in China than worldwide (Figure 1C versus 1F), indicating that bioinformatics as a field within the life sciences is enjoying a faster growth rate in China than elsewhere in the world.

These numbers and trends, although only rough estimates, are fascinating and deserve a closer look. In this article, we (see Box 1 for Authors' Biographies) take a historical, as well as a horizontal, survey of bioinformatics in China. Due to the nature of personal perspectives and space limitations, what we present is by no means exhaustive, but merely suggestive.

**Box 1. Authors' Biographies**

**Liping Wei** is Professor and Director of the Center for Bioinformatics at Peking University and Associate Director of the National Laboratory of Protein Engineering and Plant Genetic Engineering in China. She received her undergraduate training in Electrical Engineering and Information Sciences from the University of Science and Technology of China. She holds a Master's degree in Applied Mathematics from Brown University and a PhD in Medical Informatics (now called Biomedical Informatics) from Stanford University. She worked in the biotech industry for four years, first at Exelixis, Inc., and then at Nexus Genomics, Inc., while continuing to serve as a Consulting Assistant Professor of bioinformatics in the Department of Medicine at Stanford University. She moved back to China in early 2004 to resume an academic career in bioinformatics at Peking University. Her current research interests include the regulation by, and of, noncoding RNAs and antisense transcripts and the signaling and regulatory networks underlying neurobiological disorders such as autism and addiction.

**Jun Yu** is a professor in the Beijing Institute of Genomics, Chinese Academy of Sciences (CAS). Dr. Yu obtained a B.S. degree in biochemistry from Jilin University in 1983 and a PhD in biomedical sciences from New York University School of Medicine in 1990. He joined the University of Washington Genome Center in 1993 and attuned his primary research interests toward genomics and bioinformatics. He started to work in China in 1998 and has led many major genome projects there, such as the International Human Genome Project (the Chinese effort), the Superhybrid Rice Genome Project, and the Silkworm Genome Project. His current research interests include comparative genome analysis, transcriptome modeling, human genetic diversity, and model organisms for phenotypic plasticity. He has published more than 120 scientific papers and won numerous academic awards, such as Scientific Leader of the Year, 2002 (*Scientific American*), 100-Talent Plan (CAS, 2002–2005), and China–US Biology Examination and Application (CUSBEA, 1983).

## The Early Years

China had a late and rather sluggish start in bioinformatics, largely due to lack of institutional support for bioinformatics and governmental funding during the early years. It was not until 1996 that the first Center for Bioinformatics in China was established within the College of Life Sciences at Peking University and the first government funding dedicated to bioinformatics was established as part of the National High Technology Development 863 Program by the Ministry of Science and Technology (MOST). Despite the difficulties, starting from the end of the 1980s bioinformatics research was pioneered by a few scientists from other disciplines, most noticeably physics and mathematics where China's traditional strength has been, applying theoretical frameworks and analytical tools from their original specialty to study biological questions.

A great example of these early scientists was Bailin Hao, who, trained in the former Soviet Union, was at the time already an accomplished theoretical physicist. Fascinated by computable and predictable characteristics of biological systems, he first studied protein structures and then devoted himself to the analysis of DNA and protein sequences. In visualizing very long DNA sequences, including the complete genomes of several bacteria and yeast and segments of human genome, his group observed fractal-like patterns [1]; subsequently they proposed the description of a genome using statistics of *K*-strings (by counting a series of *K*-tuples in a linear sequence of symbols), enabling a new paradigm for phylogenetic analysis without sequence alignments [2]. Chunting Zhang, another established physicist, also started his bioinformatics research at the end of the 1980s working on protein structure prediction [3]. His group later created the concept of Z-curve to display DNA composition dynamics in geometric spaces [4],[5]. Z-curve has been successfully applied to genome sequence analyses such as prediction of coding genes in yeast genome [6], isochores in human genome [7], and replication origins in archaeal genomes [8]. Runsheng Chen, with a background in biophysics, was involved in the early phases of Chinese genomics research and later pioneered small RNA research in China [9]–[17]. His group identified hundreds of novel small noncoding RNAs in the nematode *C. elegans* and characterized their sequence features and expression patterns [11],[13]. A few other early scientists with important contributions include Yanda Li [18]–[24], Luhua Lai [25]–[34], Yunyu Shi [35], Liaofu Luo [36]–[40], Dafu Ding [41]–[43], and Zhirong Sun [44]–[48].

In the early to mid-1990s, many Chinese biologists were still not familiar with international biological databases, and some in remote cities did not even have reliable Internet connections to overseas Web sites. To promote bioinformatics as well as biology research, since 1995 Jingchu Luo and Xiaocheng Gu at the Center for Bioinformatics, Peking University, dedicated themselves to setting up official mirror sites of major biological databases, providing bioinformatics services, and organizing bioinformatics training workshops. From them, many Chinese scientists received their first exposure to biological data and analysis tools. Through close collaboration with the NCBI, EBI, and EMBnet, Luo's group continues to maintain the largest online bioinformatics resource in China at http://www.cbi.pku.edu.cn.

## The Internet Boom and the Genomic Boom

Two significant developments in the second half of the 1990s proved critical to the expansion of bioinformatics in China. They were the Internet boom and the genomic boom. It was not until 1994 that full TCP/IP Internet connection between China and the rest of the world was established. At that time, the only established connection was an existing link, a dial-up X.25 connection, between the Institute of High-Energy Physics (IHEP) in Beijing and the Stanford Linear Accelerator Center (SLAC) at Stanford University. On May 17, 1994, the official connection to FIX-West was announced, and the U.S.-based Energy Sciences Network (ESnet) agreed to carry China IP traffic. Despite the late start, China caught up quickly with the worldwide Internet boom and established fast and broad Internet connections which were critical for biological data exchange.

At about the same time, the genomic boom in the late 1990s to early 2000s exemplified by the Human Genome Project [49] started to generate huge and exponentially growing amounts of data. After earlier efforts such as sequencing of full-length cDNAs [50], Chinese scientists officially participated in the Human Genome Project in 1999 and sequenced 1% of the human genome [9],[49]. Since then the Chinese genome centers have independently sequenced several large genomes including those of rice (*Oryza sativa L.* ssp. *indica*) [51], silkworm (*Bombyx mori*) [52], and numerous microbial genomes such as that of *Thermoanaerobacter tengcongensis,* a rod-shaped, gram-negative, anaerobic eubacterium isolated from a freshwater hot spring in Tengchong, China [53].

Data is a major driving force for bioinformatics—it demands new methods for data storage and analyses and motivated the computational discovery of biological patterns. In all genomic projects, bioinformatics teams played significant roles in data analysis. Databases were created to store the genomic data, for instance BGI-RIS [54] and SilkDB [55] for rice and silkworm genomic sequence data and related biological information, and ChickVD [56] for chicken genomic sequence variations. Methods and software packages were developed to analyze the huge amount of genomic data, including for instance BGF, an ab initio gene prediction method developed for the rice genome based on Hidden Markov Model (HMM) and dynamic programming [57], a nonsupervised gene prediction algorithm for bacterial and archaeal genomes [58], and a method for genome comparison using Gene Ontology (GO) with statistical testing [59].

## Present Day: A Diversified Landscape

Since the end of the 1990s, bioinformatics in China has experienced significant growth fueled by not only the aforementioned Internet and genomic boom but also by official institutional support, steadily increasing government funding, and very importantly, a growing number of Chinese scientists returning to China after formal overseas training and research experience in bioinformatics and genomics. Today the field is characterized by a great variety of biological questions addressed and the close collaborations between computational scientists and bench biologists, with a full spectrum of focuses ranging from providing resources and services, developing databases and algorithms, to generating hypotheses and making biological discoveries.

Providing biological data resources and services is still an important part of bioinformatics. A good example is the Shanghai Center for Bioinformation Technology (http://www.scbit.org/), led by Yixue Li and founded in 2002. It has served as the central repository for biological data generated throughout Shanghai and neighboring areas. It has also provided extensive public online bioinformatics resources. Furthermore, many new primary databases with raw data and value-added secondary databases were created by Chinese scientists (Table 1), and many locally developed methods were implemented and incorporated into Web servers (Table 2). These databases and tools address a broad range of biological questions and are open to the entire international scientific community, and are enjoyed by a large worldwide user base.

**Table 1:** Examples of biological databases developed and maintained in China.

Category	Name	Main content	URL	Reference
**Genome resources**	BGI-RIS	An integrated information resource and comparative analysis workbench for rice genomics	http://rise.genomics.org.cn/	[54]
	SilkDB	A knowledgebase for silkworm biology and genomics	http://silkworm.genomics.org.cn	[55]
	ChickVD	A sequence variation database for the chicken genome	http://chicken.genomics.org.cn	[56]
	The Z curve database	A graphic representation of genome sequences	http://tubic.tju.edu.cn/zcurve/	[148]
	MED-Start	Predicted translation initiation sites in microbial genomes	http://ctb.pku.edu.cn/main/SheGroup/MED_Start.htm	[100]
	ProTISA	A comprehensive resource for translation initiation site annotation in prokaryotic genomes	http://mech.ctb.pku.edu.cn/protisa	[149]
	DoriC	A database of oriC regions in bacterial genomes	http://tubic.tju.edu.cn/doric/	[150]
**Transcription regulation resources**	DRTF	A database of rice transcription factors	http://drtf.cbi.pku.edu.cn/	[97]
	DATF	A database of Arabidopsis transcription factors	http://datf.cbi.pku.edu.cn	[98]
	NATsDB	A database of natural antisense transcripts identified in ten genomes	http://natsdb.cbi.pku.edu.cn/	[151]
	NONCODE	An integrated knowledge database of noncoding RNAs	http://noncode.bioinfo.org.cn	[13],[152]
	NPInter	A database of noncoding RNA and protein interaction.	http://bioinfo.ibp.ac.cn/NPInter	[14]
	ATID	A collection of publicly available alternative translational initiation events	http://bioinfo.au.tsinghua.edu.cn/atie/	[153]
	dbRES	A database for annotated RNA editing sites	http://bioinfo.au.tsinghua.edu.cn/dbRES/	[154]
	PASDB	A collection of genes reported to be alternatively spliced in plants, spanning 44 plant species	http://pasdb.genomics.org.cn	[155]
**Protein resources**	MPSS	An integrated database of protein annotations	http://www.scbit.org/mpss/	[156]
	SPD	A secreted protein database	http://spd.cbi.pku.edu.cn	[157]
	SynDB	A synapse protein database based on a synapse ontology	http://syndb.cbi.pku.edu.cn	[158]
	DBSub-Loc	Database of protein subcellular localizations	http://www.bioinfo.tsinghua.edu.cn/dbsubloc.html	[45]
	dbNEI	A database for neuro-endocrine-immune interaction.	http://bioinfo.au.tsinghua.edu.cn/dbNEIweb/	[159]
	SPIDer	Saccharomyces protein–protein interaction database	http://cmb.bnu.edu.cn/SPIDer/index.html	[130]
	InterDom	A database of putative interacting protein domains for validating predicted protein interactions and complexes	http://InterDom.lit.org.sg	[160]

**Table 2:** Examples of Web servers and software packages developed and maintained in China.

Category	Name	Main Function	URL	Reference
**Genome analysis tools**	BGF	Prediction of genes in the rice genome	http://tlife.fudan.edu.cn/bgf/	[57]
	CVTree	A phylogenetic tree reconstruction tool based on whole genome sequences without alignment	http://cvtree.cbi.pku.edu.cn	[161]
	ZCURVE	A system for recognizing protein coding genes in bacterial and archaeal genomes	http://tubic.tju.edu.cn/Zcurve_B/	[5]
	Zplotter online	A program to draw and manipulate the Z curve online based on a user's input DNA sequence	http://tubic.tju.edu.cn/zcurve/	[148]
	GS-Finder	A program to find bacterial gene start sites with a self-training method	http://tubic.tju.edu.cn/GS-Finder/	[162]
	GC-Profile	A Web-based tool for visualizing and analyzing the variation of GC content in genomic sequences	http://tubic.tju.edu.cn/GC-Profile/	[163]
	FGF	A Web tool for Fishing Gene Family in a whole genome database	http://fgf.genomics.org.cn/	[164]
	BPhyOG	An interactive server for genome-wide inference of bacterial phylogenies based on overlapping genes	http://cmb.bnu.edu.cn/BPhyOG/	[165]
	SAPRED	Using new structural and sequence attributes and Support Vector Machine to predict possible disease association of single amino acid polymorphism	http://sapred.cbi.pku.edu.cn/	[82]
**Expression regulation analysis tools**	GBA	EST-based digital gene expression profiling	http://gba.cbi.pku.edu.cn	[166]
	CEAS	An online server to analyze transcription factor binding sites based on ChIP-chip data	http://ceas.cbi.pku.edu.cn	[99]
	OTFBS	Over-represented Transcription Factor Binding Site Prediction Tool	http://www.bioinfo.tsinghua.edu.cn/7Ezhengjsh/OTFBS/index.html	[48]
	SVAP	Identification and expression analysis of alternatively spliced isoforms	http://svap.cbi.pku.edu.cn	
	CPC	Prediction of the protein-coding potential of transcripts using sequence features and support vector machine	http://cpc.cbi.pku.edu.cn	[107]
	RDfolder	A Web server for prediction of RNA secondary structure	http://rna.cbi.pku.edu.cn	[108]
	miRAS	A data processing system for miRNA expression profiling study	http://e-science.tsinghua.edu.cn/miras/	[106]
	MiPred	Classification of real and pseudo microRNA precursors using random forest prediction model with combined features	http://www.bioinf.seu.edu.cn/miRNA/	[103]
	RFRCDB-siRNA	Improved design of siRNAs by random forest regression model coupled with database searching	http://www.bioinf.seu.edu.cn/siRNA/index.htm	[167]
**Protein analysis tools**	EasyGO	Gene Ontology-based annotation and functional enrichment analysis tool for agronomical species	http://bioinformatics.cau.edu.cn/easygo/	[168]
	CTKPred	An SVM-based method for the prediction and classification of the cytokine superfamily	http://www.bioinfo.tsinghua.edu.cn/7Ehn/CTKPred/index.html	[169]
	GNBSL	A new integrative system to predict the subcellular location for Gram-negative bacteria proteins	http://166.111.24.5/webtools/GNBSL/index.htm	[127]
	MeMo	A Web tool for prediction of protein methylation modifications	http://www.bioinfo.tsinghua.edu.cn/∼tigerchen/memo/contact.html	[170]
	KOBAS	A Web-based platform for pathway identification	http://kobas.cbi.pku.edu.cn	[171]
	IntNetDB	An integrated protein–protein interaction network database generated by a probabilistic model	http://hanlab.genetics.ac.cn/IntNetDB.htm	[172]
**Platforms**	BOD	A customizable bioinformatics on demand system accommodating multiple steps and parallel tasks	http://e-science.tsinghua.edu.cn/bod/index.jsp	[173]
	ABCGrid	Application for Bioinformatics Computing Grid	http://abcgrid.cbi.pku.edu.cn	[174]

Independently or in collaboration with bench scientists, bioinformatics scientists have played significant roles in important biological discoveries. In 2003, China and many other countries suffered from the severe acute respiratory syndrome (SARS) epidemic. A team of bioinformaticians quickly joined the Chinese SARS Molecular Epidemiology Consortium, led by Guo-Ping Zhao, to sequence and analyze SARS coronavirus genomic sequences derived from the early, middle, and late phases of the SARS epidemic as well as viral sequences from palm civets. Their work uncovered the molecular evolution of the SARS coronavirus and the viral invasion from animal to human [60],[61]. Another group proposed a mathematical model to estimate the evolution rate of the SARS coronavirus genome (0.16 base/day) and the time of the last common ancestor of the sequenced SARS strains (August or September of 2002) [62]. To identify potential anti-viral drug targets, proteomic and bioinformatic methods were used to investigate key SARS viral proteins including structural proteins [63], spike protein [64], and 3C-like proteinase [30]. Genomic packaging signals, which may be used to design antisense RNA and RNA interfere (RNAi) drugs treating SARS, were predicted by comparative genomics methods [65]. The three-dimensional structure of 3C-like proteinase was constructed by homology modeling, based on which virtual screening of chemical databases was performed to search for potential inhibitors [31]. Chinese SARS patients were statistically studied to elucidate the association of symptom combinations with different outcome and therapeutic effects [66] and the association between mannose-binding lectin gene polymorphisms and susceptibility to SARS virus infection [67].

The large Chinese population provided invaluable resources for genetic studies. Patient and control groups were genotyped to establish associations between genetic variations and susceptibility to diseases such as esophageal squamous cell carcinoma [68], hepatocellular carcinoma in a particularly high-risk region of China [69], hypertension [70], and coronary heart disease [71]. A link between an Asian-enriched SNP in human sialidase and severe adverse reactions to the anti-viral drug Tamiflu (oseltamivir carboxylate) was proposed and confirmed by bioinformatic methods and enzymatic assays [72]. A number of interesting works studied the migration history of Chinese and East Asian populations by the sequencing and phylogentic analysis of Y-chromosomes [73]–[77]. Recently the Chinese HapMap Consortium participated in the international HapMap project to genotype the Chinese Han population [78]. As more and more genetic data were generated, new algorithms continued to be developed, for instance a Java-based method to analyze linkage disequilibrium [79], software for haplotype block partition and htSNPs selection [80], a method to detect human recombination hotspots based on a multiple-hotspot model and an approximate log-likelihood ratio test [81], and an SVM-based method that used new sequence and structure features to predict the possible disease association of nonsynonmous SNPs with increased accuracy [82].

The increasing number of completely and partially sequenced genomes have enabled the study of the evolution of genomes and gene families. Of particular interest to many Chinese scientists is the study of the evolution of plants, largely due to the country's longstanding strength in plant biology and partly due to its dedication to the sequencing of the rice genome. Two groups had studied the collinearity of duplicated genes along rice chromosomes and discovered that the rice genome had undergone an ancient whole-genome duplication 70 million years ago and a recent segmental duplication involving Chromosomes 11 and 12 five million years ago [83],[84]. Their findings settled previous disputes over whether rice was an ancient aneuploid or an ancient polyploidy, bringing the two contradictory sides to agreement in the form of a graciously co-authored Commentary [85]. In addition to whole genomes, the evolution of many important plant gene families was also studied in detail, such as transcription factor families [86] and enzymes [87]. A group studied the origination of new genes in rice and found a surprisingly high number of retrogenes as well as chimeric genes originated by retroposition, suggesting that retroposition is an important mechanism that governs gene evolution in rice and other grass species [88]. Another group designed a theoretical model based on the molecular clock to test the hypotheses of hybridization as an evolutionary mechanism [89]. Several groups had proposed interesting theories about the genetic code (amino acid codons and stop codons) [36],[37],[40],[90],[91].

When and where genes in a genome are transcribed and translated and how this expression of genes and proteins is regulated are important biological questions that had interested numerous scientists for decades. A first step to study this important question is to measure and compare the expression level of mRNAs. Several Chinese groups had developed algorithms for microarray experiments including designing probes [92], processing raw data by an integrated pipeline [93], detecting differentially expressed genes by relative entropy [94], identifying clusters of co-expressed genes by Hidden Markov Models [47], and finding disease-related genes by an ensemble decision approach [95]. Transcription factors bind to upstream regions of genes to regulate their transcription. About 2,000 transcription factors (∼10% of the genomes) were identified in each of the three sequenced plant genomes, *Arabidopsis*, rice, and populus, by combining similarity and motif-based approaches [96]–[98]. A new method was developed to identify potential transcription factor binding sites in the upstream regions of genes by searching for over-represented *cis*-elements with Position Weight Matrix–based similarity scores [48]. Another method uncovered the patterns of conservation, genomic distribution, and co-factor binding of transcription factor binding sites given ChIP-chip tiling array data [99]. A new four-component statistical model was proposed to improve accuracy of the identification of translation initiation sites in microbial genomes [100]. Alternative splicing adds great variety to the proteome. A new method, named Splicing Variant Analysis Platform (SVAP, http://svap.cbi.pku.edu.cn), could identify alternatively spliced isoforms from EST data ten times faster than other existing methods. Increased prediction accuracy of splice sites was achieved by using quadratic discriminant analysis with diversity measure or by introducing a competition mechanism of splice sites selection [22],[101]. The impact of very short alternative splicing on protein structures and functions was studied [21]. An interesting work identified 2,695 newly evolved exons in rodents and calculated the new exon origination rate at about 2.71×10^−3^ per gene per million years; they suggested that most new exons might originate through “exonization” of intronic sequences and appear to be alternative exons that are expressed at low levels [102].

More recently, several Chinese bioinformatics groups had made progress in studying the regulation of transcription and translation by microRNAs, noncoding RNAs, and natural antisense transcripts. New computational methods were developed to identify precursor and mature microRNAs by sequence features, hair-pin structural features, and cross-species conservations [18],[20],[103],[104]. MicroRNAs in *Chlamydomonas reinhardtii*, a unicellular green alga, were identified in reads obtained by the highly parallel 454 pyrosequencing technology; the results suggested that the miRNA pathway may be an ancient mechanism of gene regulation that evolved prior to the emergence of multicellularity [105]. To study the expression profile of microRNAs, two methods were developed using data from EST sequencing and SAGE-based total RNA clones, respectively [19],[106]. Longer noncoding RNAs were also studied extensively. Hundreds of novel noncoding RNAs were identified in *C. elegans* first by a cloning strategy and then by a whole-genome tiling array technology [11],[12]. The expression of intron-encoded noncoding RNAs was profiled using a custom-designed microarray combining noncoding RNAs and their host genes, and many noncoding RNAs were found to be independently transcribed with ncRNA-specific promoter elements [10]. To facilitate future studies, new databases were created to catalogue noncoding RNAs and noncoding RNA–protein interactions [13],[14], and new algorithms were developed to accurately predict the protein-coding potential of a given transcript and the secondary structure of an RNA [107],[108]. Many RNAs function through an antisense mechanism. Several Chinese groups have identified *cis*- and *trans*-natural antisense transcripts at the whole-genome scale and found that they are highly abundant and have interesting features of function, expression, and evolution [109]–[111].

Research at the protein level is gaining increasing momentum thanks in part to China's recognition of protein sciences as one of the major research areas to promote and support in the next 20 years. Progress continued to be made in the prediction of protein secondary and tertiary structures [46], [112]–[116]. An interesting new method achieved rapid multiple alignment of protein three-dimensional structures by the use of conformational letters, which were defined as discretized states of 3D segmental structural states [117]. Several effective scoring functions and algorithms were developed for the docking of protein with small compounds [28],[29],[33],[34]. In the field of proteomics, China joined the Human Proteome Organization (HUPO) with a special focus on the study of the human liver proteome, led by Fuchu He [118],[119]. The fetal and adult liver proteomes were extensively profiled using mass spectrometry technologies [120]–[122]. Driven by the need to identify peptides and proteins from mass spectrometry data, new algorithms were developed with increased accuracy [15], [16], [123]–[126]. Computational prediction of subcellular localizations of proteins is an important problem in proteomics, and several good methods had been developed in China using a variety of classification methods such as Support Vector Machine [44],[45], fuzzy k-NN method [23], and an integrated meta-approach [127]. Another method predicted the protein submitochondria locations by hybridizing pseudo-amino acid composition with physicochemical features of segmented sequence [24]. Protein–protein interactions were under intense study. They were predicted from sequence features [128], Gene Ontology annotations [129],[130], and integration of 27 heterogeneous genomic, proteomic, and functional annotation datasets [27]. The protein–protein interfaces were studied in detail, such as the potential of mean force used in the ranking of the binding energies of different protein–protein complexes [25] and the hydrogen bond, hydrophobic and vdW interactions used to estimate the individual contribution of each interfacial residue to the binding [26]. A recent paper reported the design of nonnatural protein–protein interaction pairs by key residues grafting [27]. The topology of the protein–protein interaction network in yeast was analyzed using a spectral method derived from graph theory to uncover hidden topological structures such as quasi-cliques and quasi-bipartites [131], and visualized as a clustering tree [17]. Metabolic pathways were identified using the KEGG Othology as an alternative controlled vocabulary [132].

Interesting progress had been made in the systems biology studies of biological networks. The global dynamical properties and stabilities of the networks had been studied. In particular, the yeast cell-cycle network and the *Drosophila* segment polarity network appeared to be highly robust [133],[134]. The protein interaction networks of *Saccharomyces cerevisiae*, *Caenorhabditis elegans*, and *Drosophila melanogaster* were found to have a scale-free and high-degree clustering nature with a small-world property with similar diameter at 4–5 [135]. Key proteins, subgraphs, and modules were identified and analyzed in the signal transduction networks [136], transcriptional regulation networks in yeast [137], and protein–protein interaction networks during fruitfly and human brain aging [138],[139]. Simulation analysis of the energy metabolism network in mammalian myocardia revealed that the systemic states of metabolic networks did not always remain optimal, but might become suboptimal when a transient perturbation occurs [140]. Finally, the dynamic properties of the arachidonic acid metabolic network which includes several targets for anti-inflammatory drugs was analyzed using ordinary differential equations, and the flux balance in the network was found to be important for efficient and safe drug design [141].

The bioinformatics discipline has grown too broad to be reviewed comprehensively in any one article. Many works cannot be covered here because of the space limitations. Just to give two examples of interesting areas of bioinformatics research that we did not adequately review here: first, a small group of researchers in China have made progress in computational neurobiology, including proposing a computational model as a neurodecoder based on synchronous oscillation in the visual cortex [142] and a simulation study on the Ca2+-independent but voltage-dependent exocytosis and endocytosis in dorsal root ganglion neurons [143]; second, many groups have developed databases and methods of imaging, such as the digital image datasets of Chinese Visible Human (a male and a female) [144], a high-resolution anatomical rat atlas [145], and methods for optical molecular imaging [146].

## Education: A New Generation of Locally Trained Bioinformaticians

Bioinformatics talent in China is reaching a critical mass. In addition to local bioinformatics scientists who converted from other disciplines and scientists who returned to China after formal training overseas, two new talent pools are forming. First, more and more scientists from other countries, especially European countries, are drawn to work in China by the improved research and funding environment. A good example is the Max Planck–Chinese Academy of Science Partners Institute in Computational Biology in Shanghai which employs a number of European scientists and plays key roles in facilitating international collaborations.

Second, a growing significant talent pool that has emerged in the past few years is the new generation of young bioinformatics scientists trained in local bioinformatics degree programs. Take the Center for Bioinformatics at Peking University as an example. By summer 2007, it had graduated 24 PhDs and four Master's degrees in bioinformatics. Although a slight majority of the graduates (15 out of 28, or 54%) still chose to pursue postdoctoral positions overseas, the remaining had decided to stay in China, with nine working in academia and four in industry. Many other Chinese universities are now offering bioinformatics degree programs at the PhD, Master's, and even Bachelor's levels (see list in Table 3). In addition to formal degree programs, many university courses and ad hoc workshops have been offered to train bench biologists on how to use bioinformatic databases and tools.

**Table 3:** Examples of bioinformatics training programs in China.

City	University/Academy	Affiliation	Degree
**Beijing**	Peking University	Center for Bioinformatics, College of Life Sciences; Center for Theoretical Biology	PhD
**Beijing**	Tsinghua University	Department of Biological Sciences and Biotechnology, Institute of Bioinformatics; Department of Automation	PhD
**Beijing**	Chinese Academy of Sciences	Beijing Institute of Genomics; Center of Systems Biology, Institute of Biophysics; Center of Molecular Systems Biology, Institute of Genetics and Developmental Biology	PhD
**Beijing**	China Agricultural University	College of Biological Sciences	PhD, Master's
**Beijing**	China Pharmaceutical University	School of Life Science and Technology	PhD, Master's
**Beijing**	Beijing Normal University	College of Life Sciences, Laboratory of Computational Molecular Biology	Master's
**Beijing**	The Academy of Military Medical Science	Institute of Basic Medical Science	Master's
**Baoding**	Hebei University	College of Life Science	Master's
**Chengdu**	Sichuan University	School of Life Sciences	PhD, Master's
**Chengdu**	University of Electronic Science and Technology of China	School of Life Science and Technology	PhD, Master's
**Chongqing**	Chongqing University of Post and Telecommunications	School of Bioinformatics	Bachelor's
**Guangzhou**	Sun Yat-Sen University	School of Life Sciences	PhD, Master's
**Hangzhou**	Zhejiang University	James D. Watson Institute of Genome Sciences, College of Life Sciences	PhD, Master's
**Harbin**	Harbin Medical University	Department of Bioinformatics	Master's
**Hefei**	University of Science and Technology of China	School of Life Sciences	PhD, Master's
**Kunming**	Yunnan University	School of Life Sciences	PhD, Master's
**Lanzhou**	Lanzhou University	School of Life Sciences	PhD, Master's
**Nanjing**	Nanjing University	School of Life Science	PhD
**Nanjing**	Southeast University	State Key Laboratory of Bioelectronics, School of Biological Science & Medical Engineering	PhD, Master's
**Shanghai**	Fudan University	Institute of Biodiversity Science, School of Life Sciences	PhD, Master's
**Shanghai**	Shanghai Institute for Biological Sciences	Key Laboratory of Systems Biology	PhD
**Shanghai**	Shanghai Jiao Tong University	Department of Biomedical Engineering, College of Life Science and Biotechnology	PhD, Master's
**Shanghai**	Shanghai University	School of Life Sciences	Master's
**Shanghai**	East China Normal University	School of Life Sciences	Master's
**Shanghai**	Tongji University	School of Life Science and Technology	PhD, Master's, Bachelor's
**Tianjin**	Nankai University	College of Life Sciences	PhD, Master's
**Tianjin**	Tianjin University	Tianjin University BioInformatics Centre, School of Science	PhD, Master's
**Xiamen**	Xiamen University	Department of Chemistry	Master's
**Wuhan**	Huazhong University of Science and Technology	School of Life Science and Technology	PhD, Master's
**Yangling**	Northwest A&F university	School of Life Sciences	PhD, Master's
**Zibo**	Shandong University of Technology	School of Life Sciences	Master's

## The Future: Promises and Challenges

Today bioinformatics research in China still lags behind the best in the world. However, it is catching up quickly, and several positive factors suggest a bright future. First, the aforementioned critical mass of bioinformaticians will be a driving force for the future development of bioinformatics in China. Second, life science research at large is gaining momentum in China and progressing quickly. Collaborating closely with talented local and international biologists, bioinformatics scientists will have access to new and numerous biological data to analyze, important biological questions to solve, and bench experimental laboratories capable of validating their predictions and hypotheses. Third, China's total funding for scientific research has been steadily increasing in the past five years, reaching 1.40% of her GDP in 2006 and a total annual R&D expenditure of 300 billion Yuan (or US$38.5 billion) [147]. The recent national goal for R&D growth has been set to an incremental annual increase of 20%. Last but not least, China is investing heavily in its computing infrastructure, including the China Grid initiated by the Ministry of Education and the China National Grid (CNGrid) initiated by Chinese Academy of Sciences, both supported largely by the Ministry of Science and Technology. Bioinformatics scientists benefit as welcomed major users of these computing infrastructures.

Despite the promising future, current challenges remain. Scientists returning from overseas often face a salary reduction, reverse culture shock, and a different funding system and application process. China lacks the equivalent of the Biomedical Information Science and Technology Initiative Consortium (BISTIC) launched at the NIH in 2000. BISTIC consists of senior-level representatives from each of the NIH institutes and centers plus representatives of other federal agencies interested in biocomputing. It plays important roles in coordinating sustained funding and other support for bioinformatics. No similar initiatives exist in China yet. There is also no official professional bioinformatics society in China. More noticeable perhaps is the lack of a Chinese national center for bioinformation, the equivalent of the US National Center for Biotechnology Information (NCBI) that is the central repository and information hub for biological data and tools. As China is generating a large and growing amount of biological data, a Chinese national center with a dedicated budget and staff could more effectively collect and store the local data and exchange it with the international scientific community. Journals and other publications in Chinese could also be collected locally and shared internationally. A Chinese national center could also offer better and faster online bioinformatics service to the large user base in China and neighboring Euro–Asian countries.

## Concluding Remarks

Bioinformatics in China has come a long way since the early years. It has benefited from developments in the rest of the world and has made its own distinct contributions. As bioinformatics in China continues to grow, we expect to see an increasing exchange of data, tools, and talent between China and other countries. We believe the future is bright for bioinformatics in China and worldwide.
